# Physiological Age Structure and *Leishmania* spp. Detection in *Phlebotomus (Larroussius) orientalis* (Parrot, 1936) (Diptera: Psychodidae) at an Endemic Focus of Visceral Leishmaniasis in Northern Ethiopia

**DOI:** 10.1155/2015/710528

**Published:** 2015-07-29

**Authors:** Araya Gebresilassie, Ibrahim Abbasi, Oscar David Kirstein, Essayas Aklilu, Solomon Yared, Habte Tekie, Meshesha Balkew, Alon Warburg, Asrat Hailu, Teshome Gebre-Michael

**Affiliations:** ^1^Department of Zoological Sciences, Addis Ababa University, 1176 Addis Ababa, Ethiopia; ^2^Department of Microbiology and Molecular Genetics, Institute of Medical Research Israel-Canada, The Kuvin Center for the Study of Infectious and Tropical Diseases, Faculty of Medicine, The Hebrew University Hadassah Medical School, 91120 Jerusalem, Israel; ^3^Aklilu Lemma Institute of Pathobiology, Addis Ababa University, 1176 Addis Ababa, Ethiopia; ^4^Department of Microbiology, Immunology and Parasitology, School of Medicine, Addis Ababa University, 1176 Addis Ababa, Ethiopia

## Abstract

Visceral leishmaniasis (VL) caused by *Leishmania donovani* is endemic in northern Ethiopia, where *P. orientalis* is the most important presumed vector. This study was designed to determine the physiological age structure and the occurrence of *Leishmania* infection in the vector of VL in Tahtay Adiyabo district, northern Ethiopia. Sand flies were collected using CDC light traps from peridomestic and agricultural fields between May 2011 and April 2012 and *P. orientalis* females were dissected for age determination and detection of *Leishmania* promastigotes. Sand flies were also analyzed for *L. donovani* detection using molecular methods. Of 1,282 *P. orientalis* examined for abdominal stages and age characterization, 66.2%, 28.2%, 4.1%, and 1.6% were unfed, freshly fed, half-gravid, and gravid. Parous rate in unfed females was 34.1% and 35.4% in peridomestic and agricultural fields, respectively. Out of 921 *P. orientalis* females dissected, one specimen (0.1%) was found naturally infected with promastigotes. Five pools (25 females) of unfed *P. orientalis* were also found with DNA of *Leishmania* spp. In particular, a single *P. orientalis* was positive for *L. donovani* (0.5%). Based on this and other evidences (abundance, human blood feeding, and xenodiagnostic studies), *P. orientalis* is the principal vector of VL in this endemic focus.

## 1. Introduction

Visceral leishmaniasis, whose causative agent is* L. donovani*, is a common parasitic infection and a major public health concern in East African countries with approximately 29,400 to 56,700 new cases each year [1]. In Ethiopia, the first case of VL was identified in the 1940s in the lower Omo plains, the southwestern part of the country [2]. Subsequently, the disease has been known to be endemic in many parts of the country. The main endemic areas foci of VL in the country include the Humera and Metema plains in the northwest [3–5], Omo plains, Aba Roba focus, and Weyto River Valley in the southwest [5–7]. However, recently increasing VL cases have been reported from previously nonendemic regions such as Libo Kemkem district of Amhara and Tahtay Adiyabo district in Tigray, northern Ethiopia [8, 9], causing high mortality and morbidity among the local residents.

The distribution of VL depends on the presence of competent vectors and reservoir hosts in a particular area. Detection of natural infection with* Leishmania* and determination of the physiological age structure of field caught sand fly vectors are of prime importance in vectorial and epidemiological studies of leishmaniasis [10]. The infection of competent vectors with* Leishmania* promastigotes has been determined by dissection of individual sand flies under a microscope. However, the use of this method to determine infection rate is difficult because promastigote infection in competent vectors is generally low and estimation of infection rate requires the examination of a large number of specimens. Previous microscopical surveys on different species of sand flies in different regions showed infection rates ranging from 1.7% to 10.7% [11–14]. Recently, polymerase chain reaction- (PCR-) based techniques have been employed to detect* Leishmania* spp. in sand flies [15–17].

In different parts of Ethiopia,* P. martini*,* P. celiae*, and* P. orientalis* have been found infected with* L. donovani* and implicated as vectors of VL [13, 18]. In particular,* P. orientalis* has been suspected as the vector of VL in the north and northwest of Ethiopia [19, 20], but it has already been incriminated in the adjacent endemic regions of Sudan [14, 21, 22]. However, data on the determination of the physiological age structure and natural infection of* Leishmania* parasites within* Phlebotomus* spp., specifically from the VL endemic area of Tahtay Adiyabo, is lacking. Therefore, the current study was carried out with the aim of determining the physiological age structure and the occurrence of* Leishmania* parasites in vector of VL in the study area.

## 2. Materials and Methods

### 2.1. Study Area and Sand Fly Collection

Field collection of sand flies was conducted in three villages (namely, Ademeyti, Lemlem, and Mentebteb) of Tahtay Adiyabo district (14°23′41′′N/37°46′15′′E) in the Tigray Regional State, northern Ethiopia. The ecology and physical environment of the study area have previously been described in detail in Gebresilassie et al. [23]. Sand flies used in the present study were originally sampled, collected, and identified as published in [23]. Briefly, female sand flies were collected from three different villages of the study area using CDC light traps between May 2011 and April 2012. Trapping of sand flies from peridomestic (places with human and animal shelters) and agricultural fields (on the periphery of settlements) was conducted for 12 nights per month. Five CDC light traps suspended with 40–50 cm above the ground level were randomly distributed to representative locations of peridomestic habitats and agricultural fields.

### 2.2. Determination of Abdominal Status, Parous Rates, and Infection Rates of Phlebotomus spp

Sand flies caught in CDC light traps from various sampling habitats were transported to a laboratory and females were transferred to test tubes. They were then knocked down using chloroform and emptied on Petri dishes for sorting into different categories (genera, sex, and abdominal status) under a dissecting microscope.* Phlebotomus* females were categorized into unfed, semigravid, gravid, and freshly fed. The freshly fed females were preserved in ethanol for parasite detection by PCR. Unfed, gravid, and semigravid female sand flies were washed in 2% savlon/saline solution followed by sterile physiological saline and dissected in sterile physiological saline for the detection of promastigotes [24]. After dissection, the ovaries of unfed* Phlebotomus* females were drawn out together with the guts to determine their parous state (parous or nulliparous). Parity was determined based on the presence of granules in the accessory glands [25] and the states of the ovaries [24]. The gut contents of parous, gravid, and semigravid females were examined under a phase contrast microscope at 40 × 10 magnifications for the presence of* Leishmania* promastigotes. When promastigotes were observed in dissected females, the gut with its content was kept in 70% ethanol and stored at −20°C for later PCR detection of* Leishmania*. Unfortunately, the promastigote positive guts could not be cultured into NNN media due to the scarcity of these media during the time of dissection.

Undissected* Phlebotomus* females were also preserved in 70% ethanol and stored at −20°C for later PCR detection of* Leishmania.* The head and abdominal tips from the above process were mounted on glass slides using Hoyer medium and identified using the identification keys of [26–28].

### 2.3. Detection of* Leishmania* Parasites by PCR

#### 2.3.1. DNA Extraction

*P. orientalis* females with abdominal status of unfed, half-gravid, and gravid were first grouped into 115 pools according to sampling date, collection site, and abdominal conditions with up to five specimens in each pool. Following this, DNA was extracted in pools from these specimens using phenol and chloroform method [9]. At the same time, DNA was processed individually from blood-fed females. Briefly, ethanol-preserved sand flies were incubated in a microfuge tube with 200 *μ*L of lysis buffer (50 mM NaCl, 10 mM EDTA, 50 mM Tris-HCl pH 7.4, 1% triton X-100, and 200 *μ*g/mL of proteinase K) at 65°C for 2 hours. Equal volumes of TE-saturated phenol (pH 8) were added to the aqueous solution; the mixture was vortexed for few seconds and then centrifuged for 2 minutes at 14,000 rpm. The upper aqueous layer was transferred to a new microcentrifuge tube and the DNA was precipitated by adding NaCl to a concentration of 0.2 M (addition of 8 *μ*L of 5 M NaCl to 200 *μ*L aqueous solution) and 2.5 volumes of 100% cold ethanol. DNA was incubated at −20°C overnight and centrifuged at 14,000 rpm for 10 minutes. The supernatant was discarded and the DNA pellet was dried in speed-vac. The DNA pellet was suspended with double distilled water and stored at −20°C until use for PCR.

#### 2.3.2. ITS Polymerase Chain Reaction (PCR)

PCR reactions were carried out in a volume of 25 *μ*L using ready mix PCR tubes (Syntezza, Jerusalem, Israel). For each reaction, 20 pmoles of the two 320 bp* Leishmania* specific ribosomal internal transcribed spacer 1 (ITS1) regions using the primers LITSR (5′-CTG GAT CAT TTT CCG ATG-3′) and L5.8S (5′-TGA TAC CAC TTA TCG CACTT-3′) was added followed by 5 *μ*L of the template DNA [29]. Double distilled water along with primers and DNA from promastigotes of* L. donovani*,* L. major*,* L. aethiopica*, and* L. tropica* was used as negative and positive controls, respectively. The thermal profile comprised 5 min at 95°C, followed by 35 cycles starting at 95°C for 30 seconds, 56°C for 30 seconds, and 72°C for 1 min, with a final elongation step at 72°C for 10 min.

#### 2.3.3. Gel Electrophoresis

Amplified products (7 *μ*L) were run on 1.5% agarose gel containing ethidium bromide for 1 h at 80 V. The gel products were visualized under ultraviolet (UV) transilluminator and then digital photographs were prepared.* Leishmania* infections were identified by comparison of PCR products of specimens with the reference strains and molecular weight markers.

*DNA Sequencing*. The amplified ITS1 PCR products that demonstrated a moderate to strong ITS1 bands were sequenced by automated fluorescent DNA sequencing using ABI PRISM 377 sequencer (PE Biosystems, Foster City, California). The sequences obtained were compared for their homology to known sequences in the GenBank database using BLAST online service provided through the PubMed/US National Institute of Health (http://www.ncbi.nlm.nih.gov/BLAST).

## 3. Results

### 3.1. Abdominal Status and Parous Rates

Tables 1 and 2 show the abdominal categories of different* Phlebotomus* spp. collected from the study villages. In Ademeyti, the number of unfed, freshly fed, half-gravid, and gravid* P. orientalis* females from peridomestic habitat was 180, 163, 17, and 2, respectively (Table 1). Similarly, more numbers of unfed (*n* = 197) and engorged (*n* = 133)* P. orientalis* were found in peridomestic biotopes of Lemlem village. Of the 60 females of* P. orientalis* obtained from Mentebteb, 40 were unfed, 17 freshly fed, and 3 gravid and half-gravid.

Of the total 524 females of* P. orientalis* dissected from agricultural fields, 430, 48, 32, and 14 were unfed, freshly fed, half-gravid, and gravid females, respectively (Table 2). Less numbers of blood-fed females were caught in agricultural fields of Ademeyti and Lemlem villages. Likewise, no blood-fed females were detected in agricultural fields of Mentebteb. However, more numbers of (*n* = 32) half-gravid females were recorded in Lemlem than the two villages.

In total, 847 unfed* P. orientalis* were dissected and examined for parous state based on the appearance of accessory glands and condition of ovaries (Table 3). Of 417* P. orientalis* dissected in peridomestic biotopes, 142 (34.1%) were parous: 33.3%, 30.9%, and 52.5% in Ademeyti, Lemlem, and Mentebteb villages, respectively, while the parous rate in agricultural fields in Ademeyti, Lemlem, and Mentebteb was 43.8%, 31.0%, and 31.4%, respectively (Table 3).

### 3.2. Natural Infection Rates with Promastigotes

Among the 921* P. orientalis* females dissected for parasite detection, one female (0.11%) was found infected with* Leishmania* promastigotes. The infected specimen was collected from agricultural field in Lemlem village during July. However, none of the other seven species of* Phlebotomus* was found infected with* Leishmania* promastigote. The number of dissected female sand flies includes 34* P. lesleyae* (1 from peridomestic and 33 from agricultural fields), 16* P. bergeroti* (12 from peridomestic and 4 from agricultural field), 6* P. papatasi* (5 from peridomestic and 1 from agricultural field), 4* P. rodhaini* (all from agriculture field), 3* P. duboscqi* (all from peridomestic), 5* P. martini* (2 from the peridomestic and 3 from agricultural field), and 2* P. heischi* (all from agricultural field).

### 3.3. *Leishmania* DNA Detection in Sand Flies

**Figure 1:**
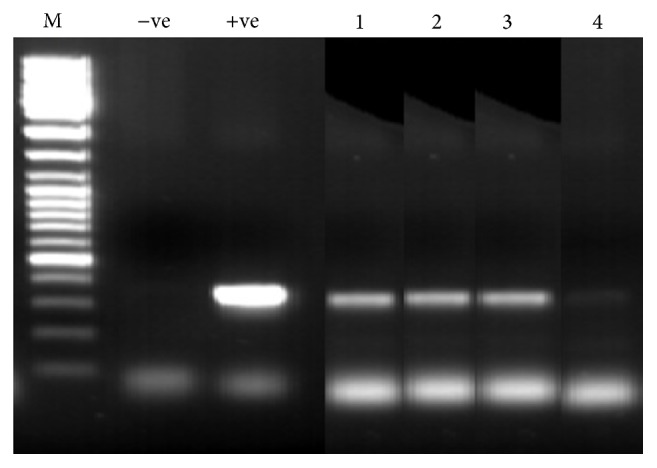
PCR of* Leishmania* internal transcribed spacer 1 (ITS1) region amplified from female sand flies. M: 100 bp size marker; −ve: negative control; +ve: reference* Leishmania* spp.; Lanes 1 and 2:* L. donovani* DNAs in* P. orientalis* and* P. bergeroti* females, respectively; Lane 3:* L. donovani* in* S. africana*; Lane 4:* Leishmania* DNA in* P. orientalis*, but failed to be sequenced.

In total, 575* P. orientalis* females, which were divided into 115 pools, were submitted to ITS1-PCR testing for* Leishmania*. Five pools were observed to possess the characteristic DNA band of* Leishmania* spp. However, acceptable sequence could not be obtained during DNA sequencing due to the weak amplification of the* Leishmania* DNA. Moreover, 200 blood-engorged sand flies (198* P. orientalis*, 1* P. bergeroti,* and 1* Sergentomyia africana*) were analyzed.* Leishmania* DNA was detected by PCR in 22 female specimens (11.0%). In the direct sequencing of the 22 PCR products, only three specimens produced a complete genomic sequence of* L. donovani* in* P. orientalis*,* P. bergeroti*, and* S. africana* females, respectively (Figure 1). Engorged specimen of* S. africana* was accidentally included in the analysis.

## 4. Discussion

Visceral leishmaniasis is affecting several people in various endemic areas of north and northwest Ethiopia. In particular, in this study area, 14.3% (680/4,757) of individuals were found positive for* Leishmania* k-DNA by real-time- (RT-) PCR [9]. In addition, 209 VL and 3 post-kala-azar dermal leishmaniasis (PKDL) cases were identified in the district in five years (2006–2011) [30].

Parous rates of* P. orientalis *dissected from peridomestic (34.1%) and agricultural field habitats (35.4%) were relatively low, which is comparable with those observed for the same species in Addis Zemen [31] and the Humera-Metema low lands [20], northern Ethiopia, and for* P. martini* in Aba Roba, southern Ethiopia [13]. The relatively small percentage of parous flies caught during the study period could indicate that females are short-lived and that few survive long enough to obtain more than one blood meal during their lives. This could also partly explain why low infection rate was detected in* P. orientalis*. Regarding the abdominal status of* P. orientalis*, large numbers of blood-fed females were sampled in peridomestic habitats of the study villages compared with agricultural fields. This higher proportion of engorged females in this habitat is largely associated with the accessibility of large numbers of blood meal sources to questing females of* P. orientalis* [32, 33]. On the other hand, relatively moderate numbers of half-gravid and gravid females were collected in agricultural fields, suggesting that older populations of the fly tend to remain in their breeding/resting sites until full development of ovules [34].

Incrimination of a certain type of sand fly vector of* Leishmania* species involves a number of criteria that include abundance in a leishmaniasis focus, high feeding habits on human, or reservoir host if the disease is zoonotic, demonstration of natural infections, and the ability to harbor, develop, and transmit the* Leishmania* parasite to a susceptible host [22, 35, 36]. Quite often, however, fulfilling all these criteria is exceedingly difficult [22, 36, 37].

Usually, a sand fly species is suspected as a vector when it is predominant and proved to have anthropophilic behavior. In this area,* P. orientalis* was found to be the preponderant (98%) and human biting* Phlebotomus* species [23]. Importantly, this sand fly species has already been incriminated as a vector of* L. donovani* in the adjacent endemic regions of Sudan [14, 21, 38] and in the Lower Omo plains of south Ethiopia [18]. The present study also detected* Leishmania* DNA by PCR in 20 blood-fed and 5 pools of unfed* P*.* orientalis* females. Specifically, one specimen of* P. orientalis* was positive for* L. donovani* after sequencing, although it was in a recently engorged female. In addition, PCR detection of* Leishmania* DNA in* P. orientalis* without blood meal (i.e., parous) is also suggestive, though sequencing of the PCR product failed to determine the exact identity of the parasites. Likewise,* Leishmania* promastigotes were detected in one specimen (1/921) of* P. orientalis* female dissected, resulting in 0.11% natural infection. The promastigotes were observed in the abdominal and thoracic midgut of the fly but with low density. Despite the positive sample yielding a characteristic DNA band of* Leishmania* in the PCR test, direct sequencing of this PCR product failed to give complete genomic sequence.

A definitive substantiation can also be drawn from the results of earlier xenodiagnosis experiment, which was carried out to demonstrate the role of asymptomatic VL persons infectious to* P. orientalis* sand flies in the area (Gebre-Michael and Hailu, unpublished). In that xenodiagnosis study, laboratory bred females of* P. orientalis* from the study area were allowed to feed on a VL-HIV coinfected patient and females were positive for promastigotes after 6-7 days of feeding. As well, the promastigotes were observed flourishing in the midgut with anterior migration to the foregut.

In addition, engorged females of* P. bergeroti* and* S. africana*, collected from Lemlem village in peridomestic habitat during January, were positive for* L. donovani* DNA.* P. bergeroti* bites human readily and has been suspected as a vector of* L. major* in the Sahara [39] and around Mecca in Saudi Arabia [40]. Different* Sergentomyia* spp. were found to be PCR positive for DNA of human pathogenic* Leishmania* species:* L. major* DNA was found in* S. darling* in Mali [17] and* S. sintoni* in Iran [41] while* L. donovani* DNA was detected in* S. babu* in the Indian VL foci [42]. However, these infections were observed by PCR methods that have no ability of discerning mature from nonmature infection. Therefore, the observations might not necessarily reflect their involvement in the transmission of the parasites to humans since* Sergentomyia* spp. do not support successful development of mammalian* Leishmania* in their guts, albeit they can be found to be PCR positive for DNA of human pathogenic* Leishmania* species [43, 44].

## 5. Conclusions

In conclusion, the predominant abundance of* P*.* orientalis* and its attraction and blood feeding habits on humans and the detection of* L. donovani* and* Leishmania* spp. in wild-caught females of* P*.* orientalis* coupled with earlier results of xenodiagnosis on a patient strongly suggest that* P*.* orientalis* is responsible for the maintenance of* L. donovani* in Tahtay Adiyabo district. Other species (e.g.,* P. martini* and* P. rodhaini*) are probably involved as secondary vectors, although they are scarce in abundance. The former is the proven vector of VL in southern Ethiopia [13] while the latter was implied as possible vector in Sudan [45]. The results presented in this paper provided baseline information on primary and suspected vectors of VL in an active focus of this disease in northern Ethiopia. These findings could be used in epidemiologic studies and strategic planning for the control of VL in the region. Further studies should be conducted to reveal the exact infection rates of* P. orientalis* in the current study area and other VL endemic foci in north Ethiopia.

## Figures and Tables

**Table 1:** Abdominal status of female *Phlebotomus* spp. captured from peridomestic sites in three different villages, May 2011 to April 2012.

Species	Number of dissected			Number of unfed			Number of freshly fed			Number of half-gravid			Number of gravid			Total
	Adm.	Lem.	Men.	Adm.	Lem.	Men.	Adm.	Lem.	Men.	Adm.	Lem.	Men.	Adm.	Lem.	Men.	
*P*. *orientalis *	362	334	60	180	197	40	163	133	17	17	2	1	2	2	2	756
*P*. *bergeroti *	3	10	1	1	3	0	2	0	0	1	2	0	1	3	1	14
*P*. *martini *	0	0	2	0	0	0	0	0	0	0	0	0	2	0	0	2
*P*. *papatasi *	2	3	0	2	3	0	0	0	0	0	0	0	0	0	0	5
*P*. *duboscqi *	2	1	0	2	1	0	0	0	0	0	0	0	0	0	0	3
*P*. *lesleyae *	1	0	0	1	0	0	0	0	0	0	0	0	0	0	0	1

Total	370	348	63	186	204	40	165	133	17	18	4	1	5	5	3	781

Adm: Ademeyti; Lem: Lemlem; Men: Mentebteb.

**Table 2:** Abdominal status of female *Phlebotomus* spp. collected from agricultural fields in three different villages, May 2011 to April 2012.

Species	Number of dissected			Number of unfed			Number of freshly fed			Number of half-gravid			Number of gravid			Total
	Adm.	Lem.	Men.	Adm.	Lem.	Men.	Adm.	Lem.	Men.	Adm.	Lem.	Men.	Adm.	Lem.	Men.	
*P*. *orientalis *	173	264	87	144	200	86	24	24	0	0	31	1	5	9	0	524
*P*. *bergeroti *	0	2	2	0	1	1	0	0	0	0	0	0	0	1	1	4
*P*. *martini *	2	1	0	1	0	0	0	0	0	1	1	0	0	0	0	3
*P*. *rodhaini *	1	3	0	1	3	0	0	0	0	0	0	0	0	0	0	4
*P*. *papatasi *	0	1	0	0	1	0	0	0	0	0	0	0	0	0	0	1
*P*. *duboscqi *	0	0	0	0	0	0	0	0	0	0	0	0	0	0	0	0
*P*. *lesleyae *	2	31	0	2	31	0	0	0	0	0	0	0	0	0	0	33
*P*. *heischi *	0	2	0	0	2	0	0	0	0	0	0	0	0	0	0	2

Total	178	304	89	148	238	87	24	24	0	1	32	1	5	10	1	571

Adm: Ademeyti; Lem: Lemlem; Men: Mentebteb.

**Table 3:** Parous rates of *P*. *orientalis* females trapped using CDC light traps, May 2011 to April 2012.

Villages	Habitat type			
	Number of dissected	Peridomestic	Number of dissected	Agricultural field
		Number of parous (%)		Number of parous (%)
Ademeyti	180	60 (33.3)	144	63 (43.8)
Lemlem	197	61 (30.9)	200	62 (31.0)
Mentebteb	40	21 (52.5)	86	27 (31.4)

Total	417	142 (34.1)	430	153 (35.6)

## References

[1] Alvar J., Vélez I. D., Bern C. (2012). Leishmaniasis worldwide and global estimates of its incidence. *PLoS ONE*.

[2] Cole A. C. E., Cosgrove P. C., Robinson G. (1942). A preliminary report of an outbreak of kala-azar in a battalion of king's African rifles. *Transactions of the Royal Society of Tropical Medicine and Hygiene*.

[3] Tekle A., Neri P., Debessai A. (1970). Kala-azar in Humera (Northwest Ethiopia). *Parassitologia*.

[4] Lyons S., Veeken H., Long J. (2003). Visceral leishmaniasis and HIV in Tigray, Ethiopia. *Tropical Medicine and International Health*.

[5] Hailu A., Gebre-Michael T., Berhe N., Balkew M., Berhane Y., Hailemariam D., Kloos H. (2006). Leishmaniasis. *Epidemiology and Ecology of Health and Disease in Ethiopia*.

[6] Ayele T., Ali A. (1984). The distribution of visceral leishmaniasis in Ethiopa. *The American Journal of Tropical Medicine and Hygiene*.

[7] Ayele T., Mekonnen M., Ali A., Hailu A. (1988). The clinical features of visceral leishmaniasis in Ethiopia. *Ethiopian Medicinal Journal*.

[8] Alvar J., Bashaye S., Argaw D. (2007). Kala-Azar outbreak in Libo Kemkem, Ethiopia: epidemiologic and parasitologic assessment. *The American Journal of Tropical Medicine and Hygiene*.

[9] Abbasi I., Aramin S., Hailu A. (2013). Evaluation of PCR procedures for detecting and quantifying *Leishmania donovani* DNA in large numbers of dried human blood samples from a visceral leishmaniasis focus in Northern Ethiopia. *BMC Infectious Diseases*.

[10] Ferro C., Morrison A. C., Torres M., Pardo R., Wilson M. L., Tesh R. B. (1995). Age structure, blood-feeding behavior, and *Leishmania chagasi* infection in *Lutzomyia longipalpis* (Diptera: Psychodidae) at an endemic focus of visceral leishmaniasis in Colombia. *Journal of Medical Entomology*.

[11] Bettini S., Gramiccia M., Gradoni L., Atzeni M. C. (1986). Leishmaniasis in Sardinia: II. Natural infection of *Phlebotomus perniciosus* Newstead 1911, by *Leishmania infantum* Nicolle 1908, in the province of Cagliari. *Transactions of the Royal Society of Tropical Medicine and Hygiene*.

[12] Maroli M., Gramiccia M., Gradoni L., Troiani M., Ascione R. (1994). Natural infection of *Phlebotomus perniciosus* with MON 72 zymodeme of *Leishmania infantum* in the Campania region of Italy. *Acta Tropica*.

[13] Gebre-Michael T., Lane R. P. (1996). The roles of *Phlebotomus martini* and *P. celiae* (Diptera: Phlebotominae) as vectors of visceral leishmaniasis in the Aba Roba focus, southern Ethiopia. *Medical and Veterinary Entomology*.

[14] Elnaiem D. A., Ward R. D., Hassan H. K., Miles M. A., Frame I. A. (1998). Infection rates of *Leishmania donovani* in *Phlebotomus orientalis* from a focus of visceral leishmaniasis in eastern Sudan. *Annals of Tropical Medicine and Parasitology*.

[15] Aransay A. M., Scoulica E., Tselentis Y. (2000). Detection and identification of *Leishmania* DNA within naturally infected sand flies by seminested PCR on minicircle kinetoplastic DNA. *Applied and Environmental Microbiology*.

[16] Pandey K., Pant S., Kanbara H. (2008). Molecular detection of *Leishmania parasites* from whole bodies of sandflies collected in Nepal. *Parasitology Research*.

[17] Berdjane-Brouk Z., Koné A. K., Djimdé A. A. (2012). First detection of *Leishmania major* DNA in *Sergentomyia (Spelaeomyia) darlingi* from cutaneous leishmaniasis foci in Mali. *PLoS ONE*.

[18] Hailu A., Balkew M., Berhe N., Meredith S. E. O., Gemetchu T. (1996). Is *Phlebotomus (Larroussius) orientalis* a vector of visceral leishmaniasis in south-west Ethiopia?. *Acta Tropica*.

[19] Gemechu T., Zerihune A., Assefa G., Lemma A. (1975). Observations of the sand fly (Phlebotominae) fauna of Setit Humera (North-Westem Ethiopia). *Ethiopian Medical Journal*.

[20] Gebre-Michael T., Balkew M., Berhe N., Hailu A., Mekonnen Y. (2010). Further studies on the phlebotomine sandflies of the kala-azar endemic lowlands of Humera-Metema (north-west Ethiopia) with observations on their natural blood meal sources. *Parasites and Vectors*.

[21] Hassan M. M., Elraba'a F. M. A., Ward R. D., Maingon R. D. C., Elnaiem D. A. (2004). Detection of high rates of in-village transmission of *Leishmania donovani* in eastern Sudan. *Acta Tropica*.

[22] World Health Organization Expert Committee (WHO) (2010). Control of the Leishmaniases. *Report of a meeting of the WHO Expert Committee on the Control of Leishmaniases Technical Report Series*.

[23] Gebresilassie A., Kirstein O. D., Yared S. (2015). Species composition of phlebotomine sand flies and bionomics of *Phlebotomus orientalis* (Diptera: Psychodidae) in an endemic focus of visceral leishmaniasis in Tahtay Adiyabo district, Northern Ethiopia. *Parasites and Vectors*.

[24] Gebre-Michael T., Pratlong F., Lane R. P. (1993). *Phlebotomus (Phlebotomus) duboscqi* (Diptera: Phlebotominae), naturally infected with *Leishmania major* in southern Ethiopia. *Transactions of the Royal Society of Tropical Medicine and Hygiene*.

[25] Añez N., Tang Y. (1997). Comparison of three methods for age-grading of female Neotropical phlebotomine sandflies. *Medical and Veterinary Entomology*.

[26] Quate L. W. (1964). Leishmaniasis in Sudan Republic. 19. *Phlebotomus* sand flies of the Paloich area in the Sudan (Diptera, Psychodidae). *Journal of Medical Entomology*.

[27] Abonnenc E., Minter D. M. (1965). Bilingual keys for the identification of the sand flies of the Ethiopian Region. *Mémoires de l'Office de la Recherche Scientifique et Technique Outre-Mer, Serie Entomologie Medicale et Parasitologie*.

[28] Gebre-Michael T., Medhin G. (1997). Morphometric separation of the females of *Phlebotomus (Phlebotomus) duboscqi* and *P. (P.) bergeroti* (Diptera: Psychodidae). *Journal of Medical Entomology*.

[29] El Tai N. O., Osman O. F., El Fari M., Presber W., Schönian G. (2000). Genetic heterogeneity of ribosomal internal transcribed spacer in clinical samples of *Leishmania donovani* spotted on filter paper as revealed by single-strand conformation polymorphisms and sequencing. *Transactions of the Royal Society of Tropical Medicine and Hygiene*.

[30] Desjeux P., Ghosh R. S., Dhalaria P., Strub-Wourgaft N., Zijlstra E. E. (2013). Report of the post Kala-Azar dermal leishmaniasis (PKDL) consortium meeting, New Delhi, India, 27–29 June 2012. *Parasites & Vectors*.

[31] Gebre-Michael T., Balkew M., Alamirew T., Gudeta N., Reta M. (2007). Preliminary entomological observations in a highland area of Amhara region, northern Ethiopia, with epidemic visceral leishmaniasis. *Annals of Tropical Medicine and Parasitology*.

[32] Gebresilassie A., Yared S., Aklilu E. (2015). Host choice of *Phlebotomus orientalis* (Diptera: Psychodidae) in animal baited experiments: a field study in Tahtay Adiyabo district, northern Ethiopia. *Parasites & Vectors*.

[33] Gebresilassie A., Abbasi I., Aklilu E. (2015). Host-feeding preference of *Phlebotomus orientalis* (Diptera: Psychodidae) in an endemic focus of visceral leishmaniasis in northern Ethiopia. *Parasites and Vectors*.

[34] Yuval B., Schlein Y. (1986). Leishmaniasis in the Jordan Valley. III. Nocturnal activity of *Phlebotomus papatasi* (Diptera: Psychodidae) in relation to nutrition and ovarian development. *Journal of Medical Entomology*.

[35] Killick-Kendrick R. (1999). The biology and control of phlebotomine sand flies. *Clinics in Dermatology*.

[36] Ready P. D. (2013). Biology of phlebotomine sand flies as vectors of disease agents. *Annual Review of Entomology*.

[37] Maroli M., Feliciangeli M. D., Bichaud L., Charrel R. N., Gradoni L. (2013). Phlebotomine sand flies and the spreading of leishmaniases and other diseases of public health concern. *Medical and Veterinary Entomology*.

[38] Hassan M. M., Elamin E. M., Mukhtar M. M. (2008). Isolation and identification of *Leishmania donovani* from *Phlebotomus orientalis*, in an area of eastern Sudan with endemic visceral leishmaniasis. *Annals of Tropical Medicine and Parasitology*.

[39] Seccombe A. K., Ready P. D., Huddleston L. M. (1993). *A Catalogue of Old World Phlebotomine Sand Flies (Diptera: Psychodidae: Phlebotominae)*.

[40] Lewis D. J., Büttiker W. (1980). Insects of Saudi Arabia, diptera family: psychodidae, subfamily: phlebotominae. *Fauna Saudi Arabia*.

[41] Parvizi P., Amirkhani A. (2008). Mitochondrial DNA characterization of *Sergentomyia sintoni* populations and finding mammalian *Leishmania* infections in this sandfly by using ITS-rDNA gene. *Iranian Journal of Veterinary Research*.

[42] Mukherjee S., Hassan M. Q., Ghosh A., Ghosh K. N., Bhattacharya A., Adhya S. (1997). Short report: *Leishmania* DNA in *Phlebotomus* and *Sergentomyia* species during a kala-azar epidemic. *American Journal of Tropical Medicine and Hygiene*.

[43] Lawyer P. G., Ngumbi P. M., Anjili C. O. (1990). Development of *Leishmania major* in *Phlebotomus duboscqi* and *Sergentomyia schwetzi* (Diptera: Psychodidae). *American Journal of Tropical Medicine and Hygiene*.

[44] Sadlova J., Dvorak V., Seblova V., Warburg A., Votypka J., Volf P. (2013). *Sergentomyia schwetzi* is not a competent vector for *Leishmania donovani* and other *Leishmania* species pathogenic to humans. *Parasites and Vectors*.

[45] Elnaiem D.-E. A., Hassan H. K., Osman O. F., Maingon R. D. C., Killick-Kendrick R., Ward R. D. (2011). A possible role for *Phlebotomus* (*Anaphlebotomus*) *rodhaini* (Parrot, 1930) in transmission of *Leishmania donovani*. *Parasites & Vectors*.

